# Inhibitory Activities of Samples on Tyrosinases Were Affected by Enzyme Species and Sample Addition Methods

**DOI:** 10.3390/ijms24076013

**Published:** 2023-03-23

**Authors:** Wei Wang, Lijuan Yang, Weiwei Wang, Jianyong Zhang, Ulrich H. Engelhardt, Heyuan Jiang

**Affiliations:** 1 Key Laboratory of Biology, Genetics and Breeding of Special Economic Animals and Plants, Ministry of Agriculture and Rural Affairs, Tea Research Institute, Chinese Academy of Agricultural Sciences, 9 Meiling South Road, Xihu District, Hangzhou 310008, China; ww1040491839@163.com (W.W.);; 2 College of Horticulture, Fujian Agriculture and Forestry University, Fuzhou 350002, China; 3 Institute of Food Chemistry, Technischen Universität Braunschweig, Schleinitzstr. 20, 38106 Braunschweig, Germany

**Keywords:** tyrosinase, species, sample addition methods, EGCG, kojic acid, β-arbutin

## Abstract

The inhibition of tyrosinase (TYR) activity is an effective measure to inhibit melanin synthesis. At present, there are many methods with discrepant details that study the TYR inhibitory activity of samples. Under the same experimental conditions, this paper systematically studies whether enzyme species and sample addition methods are the key factors that determine the TYR inhibitory activity of samples. TYRs extracted from B16F10 cells, apple and mushroom, called BTYR, ATYR and MTYR, respectively, were selected to implement this study. Results showed that TYR inhibitory activities of samples were obviously affected by the above two factors. It was necessary to select the appropriate enzyme according to the problems to be explained. It was speculated that indirectly inhibitory activity reflected the comprehensive effects of samples on TYR catalytic activity and intracellular TYR synthesis pathway, while directly inhibitory activity reflected the effects of samples on TYR catalytic activity. Additionally, kojic acid could be used as a positive control for both B16F10 cells and MTYR models. The TYR inhibitory activity of β-arbutin was complicated and fickle, while that of epigallocatechin gallate (EGCG) was universal and stable, which is to say, EGCG always inhibited TYR activity in a dose-dependent manner. In conclusion, the TYR inhibitory activities of samples were affected by enzyme species and sample addition methods. Compared with the unstable β-arbutin, EGCG was more valuable for clinical research.

## 1. Introduction

The key rate-limiting enzyme in melanin biosynthesis is tyrosinase (TYR) [1]. TYR is a metal oxidoreductase. The active site of TYR contains a coupled pair of Cu(Ⅱ) ions [2] and each Cu is coordinated by at least the Nε atoms of three histidines [3]. During the biosynthesis of melanin, TYR first acts as a hydroxylase to convert L-tyrosine to L-DOPA, and the activity of TYR at this time is usually referred to as TYR monophenolase activity. Then, TYR acts as an oxidase to convert L-DOPA into dopaquinone [4,5], which is called TYR diphenolase activity. Inhibition of TYR activity will directly lead to a decrease in the amount of newly formed melanin. Browning of fruit and vegetable, skin spots formation, UVs inducing tanning reaction, and so on, are related to the activity of TYR to some extent [5,6,7,8].

TYR is widely found in bacteria, fungi, plants and animals. TYRs have similar physiological functions in vivo, but their physical and chemical properties are different. For example, their molecular weight (about 53 kD to 75 kD of different species) and isoelectric point have a wide range of changes [9]. In addition, the nucleic acid sequence and protein molecular structure of TYR belonging to different species are different. Even in different organs and tissues of the same species, TYR has different functions [10]. The influence of samples on the activity of mushroom TYR (MTYR) [5,11] and TYR extracted from animal cells [12,13] are commonly used indicators for the preliminary screening of samples with potential anti-melanogenic activity. Based on the above species differences among TYR, we are considering whether a sample have consistent effects on enzyme activities of different species, and whether this result could be further promoted for production application or clinical research.

In addition, there are differences in the methods used to detect the effects of samples on TYR activity in animal cells. Some researchers firstly extracted TYR from mammalian cells and then reacted with the substrate (L-DOPA) in the presence of the sample. For example, Funayama et al. [14] reported that α-arbutin showed 10 times greater TYR inhibitory activity than β-arbutin in B16 mice melanoma cells. Sugimoto et al. [15] reported that α-arbutin was more effective than β-arbutin as a TYR inhibitor of HMV-II human malignant melanoma cells (L-DOPA as substrate). These results suggest that α-arbutin is a more effective skin lightener than β-arbutin. Some other researchers have used different methods to measure the effect of samples on intracellular TYR activity. In the report of Wang et al. [16], B16F10 cells were firstly cultured in the medium containing samples, during which the samples would affect the activity of TYR in cells. Then TYR was extracted from the cells and reacted with L-DOPA, which showed a stronger TYR inhibitory activity of β-arbutin compared with α-arbutin. Therefore, the effect of α-arbutin and β-arbutin on TYR (diphenolase activity) might be affected by the sample addition methods. It is worth noting that the differences in results caused by different sample addition time in the above literature are mixed with species differences of TYR to a certain extent (B16/HMV-II/B16F10 cells).

Based on these, we hope to systematically study the effects of species and sample addition methods on the TYR inhibitory activities of samples under the same experimental conditions (such as pH, temperature and time; while studying one factor, the other being fixed), because these two factors would directly affect the reliability and application range of results. B16F10 is a cell with strong ability to synthesize and secrete melanin, and it is a commonly used cell model to study the anti-melanogenic activity of samples [17]. The TYR crude enzyme extract from B16F10 cells was abbreviated as BTYR. Apple is a kind of fruit which is easily oxidized and browned. The crude enzyme, called ATYR, was extracted from apple as raw material. Additionally, MTYR was introduced as a purified commercial enzyme [18]. Kojic acid and β-arbutin, showing good TYR inhibitory activities in some researches, were commonly used as positive controls to study the anti-melanogenic activities of samples [7]. Epigallocatechin gallate (EGCG) is a key component of tea with physiological activity and wide applications [19]. The above three compounds were selected as representative samples to carry out relevant research.

In conclusion, TYRs from three sources, BTYR, ATYR and MTYR, were used to study the influence of species on the TYR inhibitory activity of samples. In terms of sample addition methods, (a) TYR extraction being followed by sample treatment and (b) sample treatment being followed by TYR extraction were designed. This study could prove the factors that might affect the TYR inhibitory activity of samples in experimental system, and provide reference for researchers who are just starting out with the anti-melanogenic experiments to select appropriate enzyme sources and experimental methods.

## 2. Results

### 2.1. Influence of Sample Addition Methods on TYR Inhibitory Activity

Two sample adding times were designed for different sample addition methods. (a) TYR extraction was followed by sample treatment. In detail, TYR was extracted from B16F10 cells or apples without sample treatment, and then mixed with samples and substrates to start the enzymatic oxidation reaction. In this process, sample only affects the catalytic activity of TYR and is in direct contact with TYR, so the effect of sample on TYR was called directly inhibitory activity; (b) Sample treatment was followed by TYR extraction. Specifically, TYR was extracted from B16F10 cells or apples treated with samples, and then mixed with substrates. Because the sample does not exist directly in the enzymatic reaction system, the effect of sample on TYR was called indirectly inhibitory activity.

**Figure 1:**
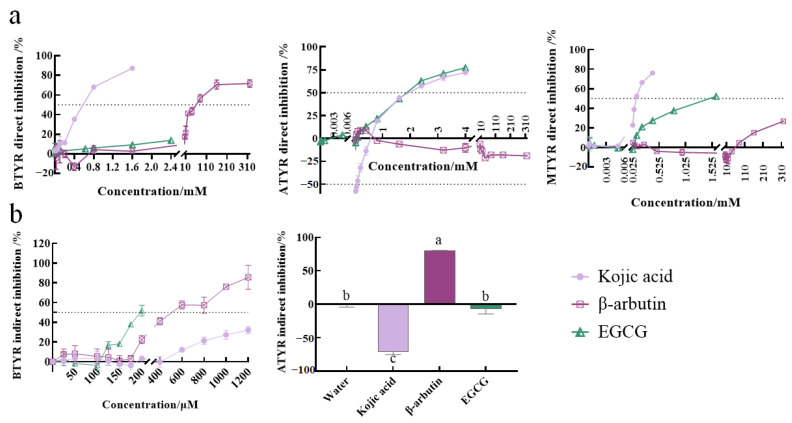
Tyrosinase (TYR) inhibitory activities of compounds were influenced by sample addition methods and enzyme species. (**a**) TYR directly inhibitory activity of compounds. After extracting enzyme solution, the enzyme, sample and substrate were mixed for enzymatic reaction in the non-cellular system. At this time, the influence of sample on enzyme activity was called TYR directly inhibitory activity. The enzymes extracted from B16F10 cells, apple and mushroom were referred to as BTYR, ATYR and MTYR, respectively. Whether the effects of compounds on TYR activity being affected by enzyme species was compared in the three subgraphs of (**a**); (**b**) TYR indirectly inhibitory activity of compounds. After sample treatment for a period of time, crude enzyme solution was extracted from B16F10 cells or apple, and then mixed with substrate for enzymatic reaction. At this time, the influence of sample on enzyme activity was called TYR indirectly inhibitory activity. ^a,b,c^ Different letters above the column indicated significant differences (*p* < 0.05).

Results showed that EGCG, kojic acid and β-arbutin inhibited the activity of BTYR in a dose-dependent manner when theses samples were in direct contact with BTYR (Figure 1a and Figure S1a). Among them, the BTYR directly inhibitory activity of kojic acid was the strongest (IC_50_ was 0.54 ± 0.02 mM). The ability of β-arbutin to directly inhibit BTYR activity (IC_50_ was 59 ± 11 mM) was only 1/100 of that of kojic acid. Although 0.025–2.4 mM EGCG directly inhibited the activity of BTYR, the inhibition rate did not reach 50%. In fact, we also studied the directly inhibitory effects of 3.2 mM EGCG and 4 mM EGCG on BTYR. However, when EGCG at above two molarities were mixed with BTYR, the reaction system becomes opaque, affecting the determination of absorbance (Figure S2). This is because EGCG, as a polyphenol, is complexed with enzyme protein at high concentration, thus affecting the chromogenic reaction between enzyme and substrate.

The effect of samples on cell viability was detected before the BTYR indirectly inhibitory experiment. Cell viability results (Table 1) showed that 25–1200 µM of kojic acid and EGCG below 200 µM did not affect cell viability. 25–1200 µM of β-arbutin enhanced cell viability in a dose-dependent manner. In the detection of indirectly inhibitory activity of BTYR, the concentrations of samples were selected as the range of non-cytotoxicity. In addition, 43.8–700 µM of β-arbutin, 175–700 µM of kojic acid and 55–218 µM of EGCG had been proved to significantly reduce melanin content of B16F10 cells in a dose-dependent manner in our previous studies [16,19]. In this study, the sample concentration with BTYR indirectly inhibitory activity almost covered the above corresponding sample concentration which could significantly reduce the intracellular melanin content. Results indicated that EGCG, kojic acid and β-arbutin indirectly inhibited the activity of BTYR in a dose-dependent manner (Figure 1b and Figure S1b). Different from the directly inhibitory activity, the indirectly inhibitory activity of EGCG (IC_50_ of 195 ± 3 µM) on BTYR was significantly stronger than β-arbutin (IC_50_ of 541 ± 20 µM), and both were stronger than kojic acid (IC_50_ was not reached at 1200 μM).

As a fruit prone to browning, apple was also used to implement related experiments in order to enhance the accuracy, reliability and commonality of results about sample addition methods. When the sample was in direct contact with ATYR in enzymatic oxidation reaction (Figure 1a and Figure S1a), EGCG inhibited the activity of ATYR in a dose-dependent manner (IC_50_ = 1.77 ± 0.02 mM). Low concentration (12.5–400 µM) of kojic acid promoted ATYR activity with EC_50_ of 0.096 ± 0.004 mM, and the lower the concentration, the stronger the promoting effect. High concentration (800–4000 µM) of kojic acid inhibited the activity of ATYR in a dose-dependent manner (IC_50_ = 2.11 ± 0.02 mM). 0.025–320 mM β-arbutin did not directly inhibit ATYR activity, but weakly promoted ATYR activity (max to 21%).

**Figure 2:**
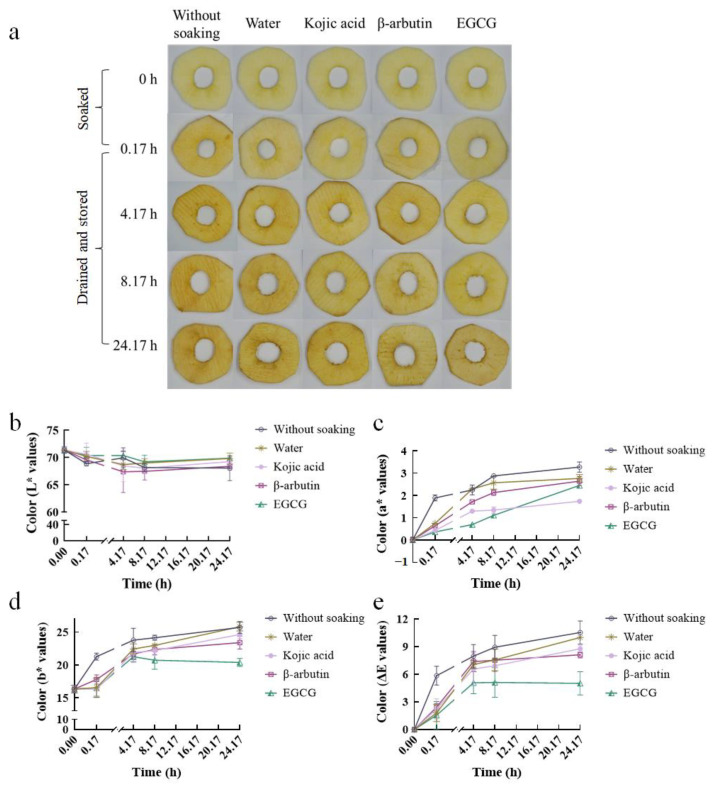
Effect of sample soaking on chromatic aberration of apple slices. Fresh apple slices of the same thickness were soaked in 1 mM of kojic acid, β-arbutin and EGCG solution for 10 min, and the change of color (**a**), L* (**b**), a* (**c**), b* (**d**) and ΔE (**e**) was observed and detected at the set times. Apple slices soaked by water were used as negative control, while apple slices without soaking were used as blank control.

Before testing the indirectly inhibitory activity of samples against ATYR, we examined whether these samples could reduce the browning of fresh-cut apple slices. The impacts of the soaking in 1 mM of EGCG, β-arbutin and kojic acid were qualitatively shown in Figure 2, where they were compared after 0.17, 4.17, 8.17 and 24.17 h from fresh cutting. Apple slices soaked by water and apple slices without soaking were used as negative control and blank control, respectively. Additionally, fresh-cut apple slices were used as the starting state of apple colorimetric measurement. As shown in Figure 2a, apple slices with various treatments gradually turned yellow, developed brown patches, lost water within 24.17 h. It means that mechanical damage, such as peeling and slicing, induced browning of apple slices in a time dependent manner and further affected shelf life of fresh-cut apples. 0.17 h after slicing, apple slices with different treatments had visible color difference, mainly between apple slices with soaking and apple slices without soaking. Apple slices with soaking had close color to that of fresh-cut apples (0 h), while apple slices without soaking were more yellow, which indicated that soaking could slow or weaken the browning of apple slices. At 4.17 and 8.17 h, the color difference among apple slices soaked by different compounds were more obvious compared with 0.17 h. EGCG had the best effect on inhibiting browning, followed by kojic acid, β-arbutin and water. The color of the slices without soaking were still the darkest. At 24.17 h, the browning degree of apple slices soaked by EGCG and β-arbutin was weaker than the other three treated apple slices. L*, a*(chromaticity on a green (−) to red (+) axis) and b* (chromaticity on a blue (−) to yellow (+) axis) are commonly used indicators to reflect color of liquid or solid. In our study, L* value hardly changed with time increasing or among different treatments (Figure 2b). a*, b* and ΔE increased in a time dependent manner (Figure 2c–e). Among them, a* has been proposed [8] might be the more discriminating one in colorimetric parameters due to an increase in a* implies an increase in redness. As shown in Figure S3b, a* of every treatment (0.17 to 24.17 h) was significantly higher than fresh-cut apple slices (0 h), indicating bruised apple browns at least ten minutes after slicing. Compared with without soaking, soaked apple slices had smaller a* value (*p* < 0.05) except for apple slices soaked by water at 4.17 h. Therefore, soaking contributed to reduce a* increasing. Compared with water, apple slices soaked by compounds could significantly weaken a* (apart for β-arbutin at 0.17 and 24.17 h), so all the three compounds could inhibit the browning of apple slices. The a* of apple slices soaked by EGCG was significantly lower than apple slices soaked by β-arbutin at 0.17 to 8.17 h, and lower than β-arbutin at 24.17 h without significant difference. There was no significant difference between EGCG and kojic acid at 0.17 and 8.17 h, while EGCG significantly lower than kojic acid at 4.17 h. These results showed that EGCG effectively reduced the increase in a* value. The b* values of each treatment had little difference (Figure S3c), but the apple slice soaked by EGCG had the smallest b* at 8.17 and 24.17 h in all treatments (*p* < 0.05). Referring to ΔE, without soaking kept the largest ΔE at all times (Figure S3d). Kojic acid had no significant difference with water at 0.17 to 24.17 h in ΔE. β-arbutin significantly reduce ΔE at 24.17 h compared with water, and EGCG significantly lower than water at 8.17 and 24.17 h. Compared with other treatments, the ΔE of EGCG was smallest in 0.17 to 24.17 h, and was close to fresh-cut apple slices. In our results, a*, b* and ΔE were able to provide a similar information that apple slices soaked by EGCG retained color variation to a minimum. At the same time, kojic acid and β-arbutin could also reduce apple slices browning to some extent through different indicators.

There are many factors affecting fresh-cut apple browning, such as temperature, oxidization and TYR. In order to minimize the influence of other factors after apple slicing up and comprehensively consider the results of chromatic aberration, apple slices soaked for 10 min were used to study the effect of samples on ATYR indirectly inhibitory activity. As showed in ATYR indirectly inhibitory activity (Figure 1b), kojic acid significantly promoted the activity of ATYR (−71 ± 4%) and β-arbutin significantly inhibited the activity of ATYR (80 ± 0%) with water (0 ± 4%) as the negative control. EGCG had no significant effect on ATYR activity (−7 ± 7%), indicating that 1 mM EGCG soaked apple for 10 min did not affect ATYR activity. Above results showed that apple slice browning was regulated by multiple factors, not completely consistent with the change of enzyme activity. In addition, only one concentration of each sample that could inhibit apple browning was selected in this experiment, so the representative on ATYR indirectly inhibitory activity in this study was slightly weak. The samples might have different inhibitory activities on ATYR under higher or lower concentration. However, at least two phenomena, 1 mM β-arbutin indirect inhibiting ATYR activity and β-arbutin direct promoting ATYR activity, were opposite.

The results of the above two enzyme sources showed that the inhibitory activities of samples on TYRs were affected by sample addition methods.

### 2.2. The Relationship between the Two Sample Addition Methods

**Figure 3:**
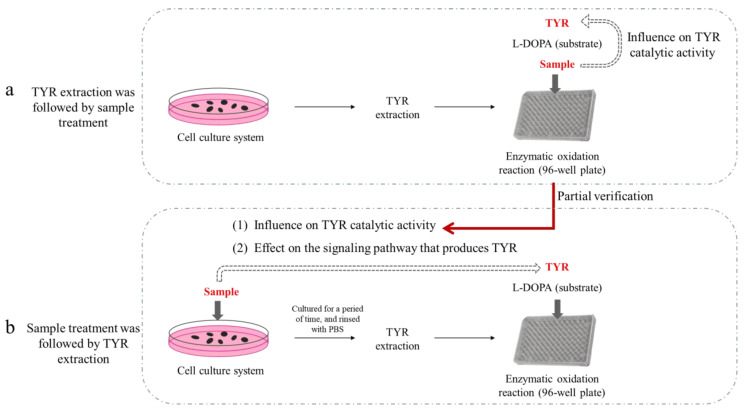
Possible influence of samples on TYR activity under different sample addition time. (**a**) TYR extraction being followed by sample treatment; (**b**) Sample treatment being follow by TYR extraction.

Possible influence of samples on TYR activity under different sample addition time was displayed in Figure 3. When TYR extraction was followed by sample treatment, sample only affects the catalytic activity of TYR (Figure 3a). As shown in Figure 3b, the influence of sample on TYR activity during this process may represent the effect of sample on TYR catalytic activity (the premise being that the sample can enter into the cell and come into contact with enzymes inside the cell), and may also be an effect on the signaling pathway that produces TYR (the synthesis of new TYR being affected). Keeping the concentration of sample consistent in both sample addition methods, if the influence on catalytic activity is excluded, the influence of sample on signaling pathway can be further studied. Therefore, the two methods might be used together to illustrate some problems to some extent.

**Figure 4:**
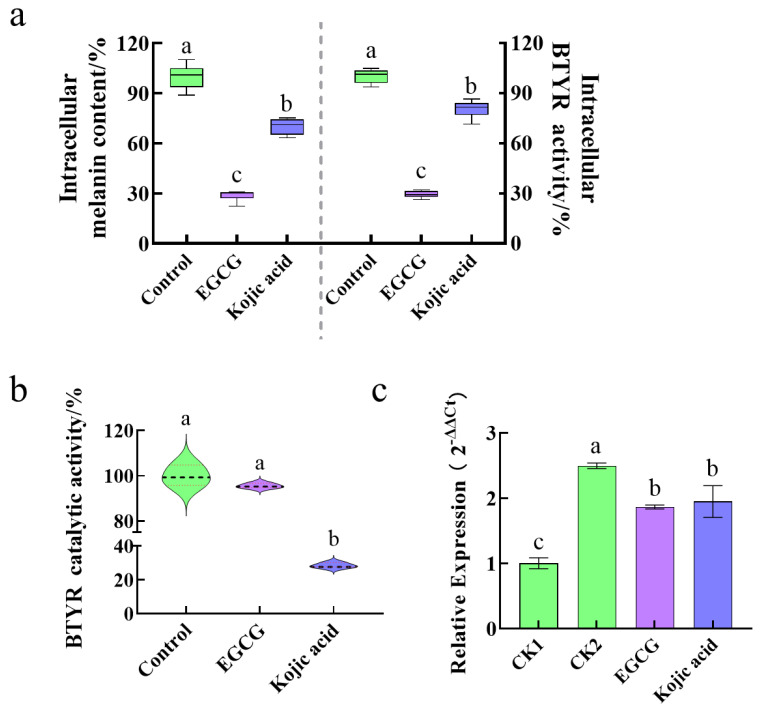
The mechanism of 100 μg/mL EGCG and kojic acid on inhibiting BTYR activity. (**a**) Influence of EGCG and kojic acid on intracellular melanin content and BTYR activity. α-MSH stimulated B16F10 cells were cultured by complete medium containing compounds for 48 h. Then intracellular melanin content and BTYR activity were detected; (**b**) BTYR catalytic activity of EGCG and kojic acid. TYR extraction was followed by sample treatment. The reaction among BTYR, substrate and sample was conducted in non-cellular system; (**c**) Effect of EGCG and kojic acid on expression of BTYR mRNA. α-MSH stimulated B16F10 cells were cultured by complete medium containing compounds for 24 h. Then the expression of BTYR mRNA was detected. CK1 was medium alone-added group, while CK2 was α-MSH alone-stimulated group. ^a,b,c^ Different letters above the plots indicate significant differences (*p* < 0.05).

In order to verify the above hypothesis, 100 μg/mL EGCG (218 μM) and kojic acid (704 μM) were used as samples, and BTYR was selected as the target enzyme. The effects of two samples on intracellular BTYR activity, catalytic activity of BTYR in non-cellular system and intracellular BTYR mRNA expression level were analyzed. As shown in Figure 4a, EGCG and kojic acid could significantly reduce intracellular melanin content and inhibit intracellular BTYR activity, which further confirms the results in Figure 1b. It is worth noting that 100 μg/mL kojic acid could significantly inhibit the catalytic activity of BTYR, while EGCG could not (Figure 4b). However, both EGCG and kojic acid could significantly inhibit the mRNA expression of BTYR in cells after cultured by samples for 24 h (Figure 4c). The above results indicate that EGCG and kojic acid inhibit BTYR activity by different mechanisms. It can be summarized that 100 μg/mL EGCG could not reduce BTYR catalytic activity, but does inhibit intracellular BTYR activity through down-regulating BTYR mRNA expression. The results of EGCG fully characterize the differences of the two sample addition methods and the implications of their combination. The results for kojic acid are more complicated and interesting. It was speculated that 100 μg/mL kojic acid inhibited intracellular BTYR activity by down-regulating BTYR mRNA expression and reducing BTYR catalytic activity.

Although kojic acid significantly inhibited the catalytic activity of BTYR compared with EGCG and no difference was found between EGCG and kojic acid on TYR mRNA expression, the ability of kojic acid to inhibit intracellular BTYR activity was significantly lower than EGCG. This phenomenon indicated that the influence of samples on intracellular physiological metabolism is very complex, including not only TYR synthesis, but also possibly TYR degradation and other processes. This provides a path for researchers to carry out more in-depth research, that is study extending from effective combination of two sample addition methods.

### 2.3. Effects of Enzyme Species on TYR Inhibitory Activity of Samples

In addition to sample addition methods, the species of enzyme may be another factor affecting the TYR inhibitory activity of the sample. Firstly, whether TYR inhibitory activity of samples being influenced by TYR species was investigated when samples were in direct contact with TYR in enzymatic oxidation reaction. As shown in Figure 1a, large sample concentration ranges (320 to 100,000 times) were set to study as fully as possible whether there was differences in the activities of these samples at high and low concentrations. When the directly inhibitory activities of BTYR, ATYR and MTYR were studied, the molarities of kojic acid were 0.04–1600 μM, 12.5–4000 μM and 0.04–400 μM, respectively. The molarities of β-arbutin were 0.025–320 mM, 0.025–320 mM and 0.03–320 mM, respectively. The molarities of EGCG were 0.04–2400 μM, 0.04–4000 μM and 0.04–1600 μM, respectively. The result indicated that kojic acid had dual effects on ATYR activity. Low concentration of kojic acid promoted ATYR activity, while high concentration of kojic acid inhibited ATYR activity. Dual effects of kojic acid were not found in BTYR and MTYR activity. Kojic acid inhibited the activities of BTYR and MTYR in a dose-dependent manner. With the decrease in kojic acid concentration, the inhibitory effect on BTYR and MTYR activity was infinitely close to zero. Although TYR activity fluctuated somewhat treated by β-arbutin at lower concentrations (0.025–0.8 mM), β-arbutin inhibited BTYR activity in a dose-dependent manner at higher concentrations (0.8–320 mM). β-arbutin dose-dependently and slightly prompted ATYR activity. 0.48 to 40 mM β-arbutin slightly promoted MTYR activity (4% to 13%), while 80 to 320 mM β-arbutin inhibited MTYR activity in a dose-dependent manner (5% to 27%). It is interesting to see the transition of the effect of β-arbutin on MTYR activity after reaching a certain concentration, although the degree of promotion and inhibition was relatively weak. EGCG could inhibit the activity of BTYR, ATYR and MTYR in a dose-dependent manner. When the concentration of EGCG was reduced, the inhibitory effect of EGCG on TYR activity was infinitely close to zero, but it did not promote the activity of TYR. In above three compounds, at least the results of kojic acid and β-arbutin had proved that the TYR species affected the effects of those specific compounds on TYR activity.

Except for the compounds showed either promoting or inhibiting activity in different species of enzymes, there were also species differences in sample activity comparison. As shown in Figure 1a, the MTYR directly inhibitory activity of EGCG (IC_50_ of 1421 ± 61 μM) was only 1/15 of that of kojic acid (IC_50_ of 94 ± 1 μM), which was consistent with the result that EGCG was weaker than kojic acid in the directly inhibitory activity of BTYR. While in ATYR directly inhibitory activity, EGCG was stronger than kojic acid.

Secondly, there were also species differences in the TYR indirectly inhibitory activities of samples (Figure 1b). The three samples all indirectly inhibited the activity of BTYR in a dose-dependent manner. The inhibitory activity of EGCG was stronger than that of β-arbuin, and both of them were stronger than kojic acid. However, in the indirectly inhibitory activity of ATYR, 1 mM EGCG did not affect the activity of ATYR, 1 mM β-arbutin significantly inhibited the activity of ATYR, and 1 mM kojic acid significantly promoted the activity of ATYR. That was, there were species differences in the comparison of indirectly inhibitory activity between EGCG and β-arbutin, while kojic acid showed opposite activity in the two species.

In summary, the TYR inhibitory activity of the samples was affected by the enzyme species.

### 2.4. Kinetic Analysis

We were not able to obtain purified TYR extracted from apple or B16F10 cells so as to further study the inhibition types of these enzymes treated by samples. Therefore, this part was mainly carried out for commercial MTYR. The effect of β-arbutin on the directly inhibitory activity of MTYR was fickle (Figure 1a). β-arbutin directly promoted MTYR activity (lower than 80 mM) and then inhibited MTYR activity (greater than 80 mM) in a dose-dependent manner. In our previous study [16], we failed to identify the inhibitory type of β-arbutin on MTYR. Compared with β-arbutin, EGCG and kojic acid showed more simple effects on MTYR activity. That was, both of them directly inhibited MTYR activity in a dose-dependent manner. Therefore, this study mainly studied the inhibitory types and inhibitory constants of EGCG and kojic acid on MTYR.

**Figure 5:**
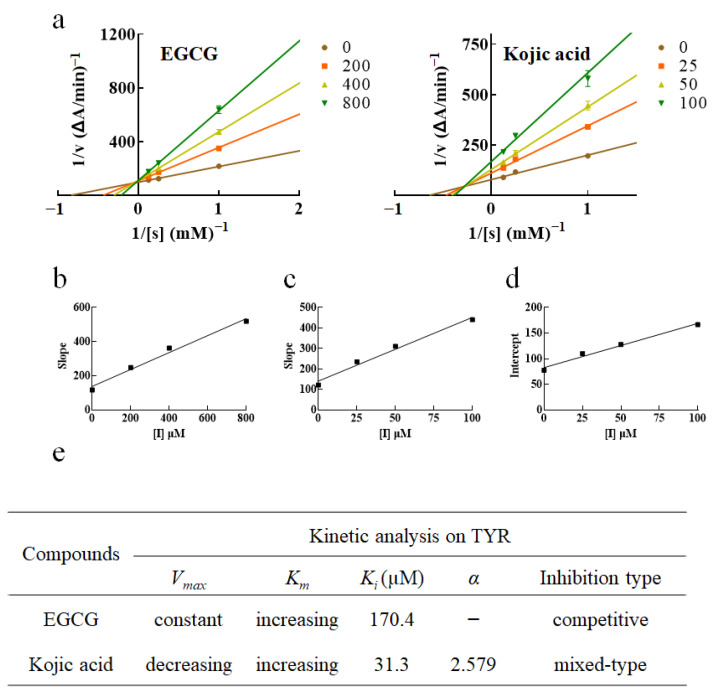
Inhibitory types and inhibitory constants of kojic acid and EGCG on MTYR: (**a**) Lineweaver–Burk plots on MTYR of EGCG and kojic acid; (**b**) Secondary replot of slopes (Lineweaver–Burk plot) vs various concentrations of EGCG; (**c**,**d**) Secondary plots of slopes and intercept (from Lineweaver–Burk plots) vs various concentrations of kojic acid; (**e**) Kinetic parameters and inhibition type of EGCG and kojic acid. Changes of *V_max_* and *K_m_* with the increasing compound molarity were shown. The substrate in the enzymatic reaction was L-DOPA.

In the analysis of enzyme kinetics, a series of different concentration of samples and substrate (L-DOPA) were set up to obtain the initial rate of enzymatic reaction of each treatment. 1/V against 1/[S] was plotted to get a series of lines with different slopes. The results showed that *K_m_* increased with increasing concentration of EGCG, but *V_max_* remained unchanged. The intersection of lines was on the Y-axis. Based on these results, it was speculated that EGCG was a competitive inhibitor of MTYR (Figure 5a,e). With the increase in kojic acid concentration, *K_m_* increased and *V_max_* decreased, and the intersection of the lines was in the second quadrant, indicating that kojic acid was a mixed (competitive-noncompetitive) inhibitor of MTYR (Figure 5a,e) [20].

Inhibition constant, called *K*_i_, was calculated by replot of slopes (Lineweaver–Burk plot) vs various molarities of inhibitor [21]. *K*_i_ is relevant to inhibitory activity of inhibitor, being the smaller the value of *K*_i_ the stronger is the inhibitory activity [8]. *K*_i_ values of EGCG and kojic acid were 170.4 μM (Figure 5b,e) and 31.3 μM (Figure 5c,e) respectively. Therefore, when the compounds were in direct contact with MTYR, kojic acid possessed stronger inhibitory activity against MTYR than EGCG, as uncovered by the values of IC_50_.

Kojic acid, as a mixed inhibitor, not only interacts with free enzymes, but also with enzyme-substrate complexes. Alpha (α) determines the mechanism, and its value decides the degree to which the binding of inhibitor alters the affinity of the enzyme to the substrate. α of kojic acid was 2.579 (Figure 5d,e), which meant that kojic acid preferentially binds to the free enzyme rather than enzyme-substrate complex (When α > 1, the inhibitor preferentially binds to the free enzyme).

In addition, slope or intercept was replotted versus sample concentrations (Figure 5b–d), which showed good linear fitting, indicating that EGCG and kojic acid had a single or a class of inhibition sites for MTYR [20].

## 3. Discussion

TYR is a key rate-limiting enzyme in melanin biosynthesis. Inhibition of TYR activity is an effective measure to reduce the formation of melanin, and is also a common indicator to screen compounds with anti-melanogenic activity. This study mainly explored some interesting and contradictory phenomena about enzyme activity founded in previous studies, that is, whether the species of enzyme and the sample addition methods were the influencing factors for the TYR inhibitory activity of sample.

The TYR inhibitory activity of sample was affected by sample addition methods. In the directly inhibitory activity of BTYR, all of EGCG, kojic acid and β-arbutin, inhibited the activity of BTYR in a dose-dependent manner, however, the activity of kojic acid was stronger than that of EGCG and β-arbutin. In the indirectly inhibitory activity of BTYR, although EGCG, kojic acid and β-arbutin indirectly inhibited BTYR activity in a dose-dependent manner, the activities of EGCG and β-arbutin were stronger than that of kojic acid. When using ATYR as an enzyme source, 0.025–320 mM β-arbutin directly promoted the activity of ATYR, while 1 mM β-arbutin significantly and indirectly inhibited the activity of ATYR, showing a completely different action of mode. This was a thought-provoking and interesting result. Lim et al. [13], in order to ascertain the anti-melanogenic mechanism of SNA077, explored the direct TYR inhibitory activity of sample using MTYR, while measured intracellular TYR activity by using TYR extracted from α-MSH-stimulated and sample-treated B16 cells. They found that SNA077 did not inhibit MTYR activity, but inhibit the activity of B16-derived TYR, which was concluded that the anti-melanogenic mechanism of SNA077 was derived from reducing the gene expression level of TYR rather than affecting direct TYR inhibitory activity. The two experimental methods Lim et al. [13] used, could reflect the mechanism how the sample affected the activity of TYR (such as influencing the catalytic activity of enzymes or regulating the expression of related genes and proteins in cells), but the species of enzyme probably should be the same in both methods in order to control unique variable. That was, the detecting of direct TYR inhibitory activity might be more suitable if using TYR extracted from B16 cells without treated by samples as the source. The essential difference between direct or indirect inhibition is the way in which the sample acts on the enzyme. The results of direct inhibition mainly reflect the influence of the sample on the spatial conformation of enzyme and the binding force between enzyme and substrate, which could be further verified by enzyme kinetic analysis and molecular docking experiments. The results of indirect inhibition reflect the comprehensive effects of samples on cell physiological metabolism, gene or protein expression in signaling pathway, enzyme catalytic activity, etc. According to the problems to be explained, it is necessary to select a suitable experimental method to determine the effect of the sample on the enzyme activity. For example, when studying the effect of compounds on the binding of enzymes to substrates, sample being in direct contact with TYR in enzymatic oxidation reaction is appropriate. When it is necessary to find intracellular signaling pathways that influence enzyme activity, sample treatment being followed by TYR extraction can be used. In some cases, two sample addition methods are more suitable for being used together to illustrate some problems, such as the BTYR inhibitory activity displayed by EGCG and kojic acid in Figure 4.

It was found that the TYR inhibitory activity of samples was also affected by enzyme species. For example, (1) in direct inhibition, kojic acid inhibited BTYR and MTYR activities in a dose-dependent manner. With the decrease in kojic acid concentration, its inhibitory activity on BTYR and MTYR was close to zero. Kojic acid has a double effect on ATYR activity. Low concentration of kojic acid promoted ATYR activity, while high concentration of kojic acid inhibited ATYR activity. (2) In direct inhibition, the effects of β-arbutin at lower concentration (0.025–0.8 mM) on BTYR activity were unstable, while higher concentration (0.8–320 mM) of β-arbutin inhibited BTYR activity in a dose-dependent way. β-arbutin slightly increased ATYR activity in a dose-dependent manner (the maximum increase was only 21%). 0.48–40 mM of β-arbutin weakly promoted MTYR activity (4%–13%), whereas 80–320 mM of β-arbutin dose-dependently inhibited MTYR activity (5%–27%). (3) In the directly inhibitory activity of MTYR and BTYR, EGCG was weaker than kojic acid. In the directly inhibitory activity of ATYR, EGCG was stronger than kojic acid. (4) In indirect inhibition, kojic acid inhibited the activity of BTYR in a dose-dependent manner, but 1 mM kojic acid significantly increased the activity of ATYR. In addition, which activity of EGCG or arbutin was stronger was affected by species. Similar contradictory results have been reported in the previous literatures. Funayama et al. [14] found that α-arbutin could not inhibit the diphenolase activity of MTYR, while β-arbutin could (IC_50_ was 8.4 mM), but as a TYR diphenolase inhibitor in B16 mouse melanoma cells, α-arbutin was 10 times more potent than β-arbutin. In other words, α-arbutin and β-arbutin showed different inhibitory activities in TYR of different species. Mann et al. [22] thought that the clinical efficacy of currently used TYR inhibitors was unsatisfactory, largely because these compounds were tested using only TYR isolated from mushroom Agaricus bisporus. It was suggested that a compound might have differently inhibitory activity on mushroom or human TYR (HTYR). Although being effective against MTYR, most TYR inhibitors turned out to be poor inhibitors of HTYR. The substrate specificity and catalytic activity of MTYR have been proved to be significantly different from the mammalian TYR [23]. For example, the activity of hydroquinone on HTYR (IC_50_ = 4400 mM) was 1/4000 of that on MTYR (IC_50_ = 1.1 mM) [22,24]. In fact, species differences are not uncommon. People, mice and rats have different sensitivity to APAP-induced hepatotoxicity [25]. For the same traditional Chinese medicine (TCM), experimental animals of different species differ greatly in TCM efficacy, toxicity and metabolism [26]. Liu Shanting et al. [27] found that the cortex sophorae extract had obvious inhibitory effect on the duodenum of rabbits, rats and mice, but had excitatory effect on the duodenum of guinea pigs. The differences between species may be due to the different structures of TYR active centers from different sources. Therefore, the inhibitory effect of samples on TYR of one species cannot be directly used to judge the influence of samples on TYR activity of another genus. The TYR species may interpret what the results tell us. The results are often referred to as anti-melanogenic activity, but this activity is not specific, and can be further subdivided into activities such as inhibiting fruit and vegetable browning, whitening, and inhibiting UV-induced skin tanning. Therefore, we speculated that the results of plant-derived TYR might be more able to explain the problem of fruit and vegetable browning, while animal-derived TYR might be more able to explain the latter two phenomena. Of course, the results of enzymes should be combined with other indicators to illustrate the activity of the samples. For example, both chromatic aberration and TYR activity should be tested when studying the activity of samples to inhibit fruit and vegetable browning. In terms of inhibiting intracellular melanin synthesis, cell viability, melanin content and TYR activity supplement each other. Considering timeliness of massive sieve samples, animal welfare and ethics, it is more economical, fast and easy to realize high-throughput screen to study TYR inhibitory activity of samples by using MTYR and animal-derived TYR as enzyme source. However, the species difference is an inevitable challenge to extrapolate the results from non-cellular systems, cellular systems and animal experiments to humans. Fully understanding the differences between species, and then selecting appropriate enzyme sources for research, can provide a better scheme for screening potential TYR inhibitors in the future.

In this study, CCK-8 method was used to determine the changes of cell viability under each treatment, and cell viability was used to represent cell survival rate, thus indicating the toxicity of samples to cells. In our previous study [16], 43.8–700 μM α-arbutin and β-arbutin not only dose-dependently inhibited TYR activity and decreased melanin content, but also dose-dependently increased cell viability, especially in β-arbutin. In this research, the results of cell viability of β-arbutin also showed a consistent phenomenon. However, some results on cell proliferation rate obtained by cell counting method showed that when the concentration was lower than 1 mM, α-arbutin did not inhibit the growth of HMV-II after α-arbutin culture [28]. 100 μg/mL (367 μM) β-arbutin also did not affect the proliferation of human melanoma cells and normal melanocytes cultured by medium containing samples, but 300 μg/mL (1102 μM) β-arbutin was cytotoxic [29]. The cell proliferation described above meant the change in the number of cells. Another experiment showed that after treated by 0.5–8 mM β-arbutin for 3 days, the number of normal melanocyte cells decreased [30]. CCK-8 and cell counting are both commonly used methods to detect the cytotoxicity of samples, but their principles are different. The primary component in CCK-8 is a water-soluble tetrazole salt (WST^®^-8). The orange-yellow formazan dye obtained by the oxidative reduction of WST^®^-8 triggered by intracellular dehydrogenase is able to dissolve in the medium. The amount of formazan dye generated is proportional to the number of living cells [31]. Therefore, CCK-8 indirectly reflects cell number with cell viability, while cell count is an accurate count of the increase or decrease in cell number after cultured by samples. CCK-8 may not be able to accurately represent the change of cell number after sample treated. Of course, even if there is no significant change in the number of cells treated by sample, the intracellular physiological activity has been drastically changed in some cases, as shown by β-arbutin. These results suggest that CCK-8 and cell counting complement and verify each other. More parallel experiments would be needed to explore the cause of a significantly increase in cell viability, even though there is no difference in cell number. In a report obtaining cell proliferation results by using CCK-8 assay, 12.5–200 μM β-arbutin did not affect cell viability [32]. The difference between this above result and our results may be caused by different cells (B16/B16F10), whether the cells were induced by α-MSH (not induced by α-MSH/induced by α-MSH), different culture time (24 h/48 h), and even different medium formula. Kojic acid (25–1200 µM) and EGCG (25–200 µM) consistently did not affect cell viability in our study. EGCG above 200 µM reduced cell viability in a dose-dependent manner, which was consistent with the changes in cell number observed under the microscope. Therefore, more methods were needed to verify the effect of β-arbutin on cell viability and to explore the causes in the future.

In this experiment, kojic acid had a double effect on ATYR activity. Low concentrations (12.5–400 µM) of kojic acid promoted ATYR activity, while high concentrations (800–4000 µM) of kojic acid inhibited it. Similar results have been found in the previous literature. Pyrogallol is an organic gallic acid conversion compound with three hydroxyl groups and belongs to the phenol family [33]. When pyrogallol exists alone in the reaction system, it is the substrate of TYR [34,35]. When pyrogallol and L-DOPA appeared in the reaction system at the same time, pyrogallol was not the substrate of TYR, but the inhibitor of L-DOPA [33]. This phenomenon is not uncommon. For example, flavonoid compounds such as quercetin are considered to be inhibitors and substrates of TYR [36,37]. High-dose pyrogallol inhibited TYR activity, while low-dose pyrogallol enhanced TYR activity [33]. The activation of TYR activity by low concentration inhibitors has long been reported [38,39,40], mostly believed to be related to the conformational flexibility of the active site of TYR and the function of copper co-catalytic oxidation. Whether kojic acid exhibits dual effects on ATYR activity through the same mechanism (inhibitor and substrate of TYR) remains to be further investigated.

In addition, the results of inhibition types showed that EGCG was a competitive inhibitor of MTYR diphenolase activity, while kojic acid was a mixed inhibitor of MTYR diphenolase activity. *K*_i_ results showed that kojic acid had stronger inhibitory activity against MTYR than EGCG when the compound was in direct contact with MTYR, further confirming the results of IC_50_.

In this study, two conceivable and potential factors, enzyme species and sample addition methods, that might affect TYR inhibitory activity of samples were systematically studied under unified experimental conditions, which can help the researchers who are new to anti-melanogenic activity to avoid traps and carry out more valuable research faster. At the same time, it was found that EGCG had universal and stable TYR inhibitory activity, which might have a better clinical study value compared with the unstable β-arbutin.

## 4. Materials and Methods

### 4.1. Reagents

Dulbecco’s modified Eagle’s medium (DMEM), α-MSH (A1025), kojic acid (≥99%), 3,4-dihydroxyphenylalanine (L-DOPA), and MTYR were obtained by the same way as our previous literature [16,19].

Fetal bovine serum (FBS) was purchased from Gibco (Grand Island, NE, USA). D-PBS, cell counting kit-8 (CCK-8), dimethyl sulfoxide (DMSO) and P0013J lysis buffer were purchased from Beyotime (Shanghai, China). NaOH was purchased from Sinopharm chemical reagent Co., Ltd. (Shanghai, China). EGCG (98%) was a generous gift from the Tea Research Institute of Chinese Academy of Agricultural Sciences. β-arbutin (≥99%) and polyvinylpolypyrrolidone (PVPP) were purchased from Shanghai Aladdin Biochemical Technology Co., Ltd. (Shanghai, China).

### 4.2. BTYR Directly Inhibitory Activity of Compound

B16F10 cells at the exponential phase in 10 cm^2^ cell culture dish were washed with D-PBS two times and incubated for 30 min at −20 °C in 1.16 mL of lysis buffer. The lysates, called BTYR, were centrifuged at 12,000 rpm for 10 min at 4 °C.

The reaction system in 96-well plate was 100 μL. 20 μL of BTYR was mixed with 20 μL compounds and incubated at 25 °C for 10 min, then 60 μL of 8 mM L-DOPA was added to start the reaction. A Synergy H1 microplate reader (BioTek Instruments, Inc., Winooski, VT, USA) was used to dynamically monitor the optical densities at 475 nm. Absorbance was measured every minute and sustained 30 min. The initial velocity (*v*_0_) of each reaction was represented by the slope of the initial linear range of the progress curve, and was used to calculate the relative enzyme activity of each treatment [16].

The molarity of kojic acid was 0.00004, 0.0002, 0.001, 0.005, 0.0125, 0.025, 0.05, 0.1, 0.2, 0.4, 0.8 and 1.6 mM. The molarity of β-arbutin was 0.025, 0.05, 0.1, 0.2, 0.4, 0.8, 1.6, 3.2, 5, 10, 20, 40, 80, 160 and 320 mM, while that of EGCG was 0.00004, 0.0002, 0.001, 0.005, 0.025, 0.125, 0.625, 0.8, 1.6 and 2.4 mM.

BTYR directly inhibitory activity compared to negative control could be expressed by following formula [16].

Δsample = sample − sample control, Δcontrol = negative control − blank control(1)

k_Δsample_ = (Δsample_t1_ − Δsample_t0_)/(t_1_ − t_0_), k_Δcontrol_ = (Δcontrol_t1_ − Δcontrol_t0_)/(t_1_ − t_0_) (2)

(3) $$\mathrm{BTYR}\;\mathrm{direct}\;\mathrm{inhibition}\;\left(\%\right)=\left(1-\frac{k_{\Delta \mathrm{sample}}}{k_{\Delta \mathrm{control}}}\right)\times 100$$

Note: Sample group (there are BTYR, compound, and L-DOPA); Sample control group (there are BTYR and compound, but no L-DOPA); Negative control group (there are BTYR and L-DOPA, but no compound); Blank control group (there are TYR only).

### 4.3. BTYR Indirectly Inhibitory Activity of Compound

#### 4.3.1. Cell Culture

Referring to our previous methods [16]. In short, B16F10 murine melanoma cells from Stem Cell Bank (Chinese Academy of Sciences, Shanghai, China) were cultured in DMEM supplemented with 10% FBS in a humidified atmosphere of 5% CO_2_ at 37 °C.

#### 4.3.2. Measurement of Cell Viability

Measurement of cell viability was determined according to our previous procedure [19]. In brief, cells were seeded into 96-well plates at a density of 1 × 10^4^ cells/well and incubated for 24 h. Then, the culture medium was replaced with complete medium containing compound and further incubated at 37 °C for 48 h. After rinsing once with D-PBS, 100 µL DMEM containing 10% CCK-8 was added to each well. Followed by another 1 h incubation, the absorbance at 450 nm was measured.

The molarities of kojic acid, β-arbutin and EGCG were 25, 50, 100, 200, 400, 600, 800, 1000 and 1200 μM. The cell viability was expressed as a percentage of the control.

#### 4.3.3. Measurement of Intracellular Melanin Content

Measurement of intracellular melanin content was determined according to our previous procedure [19]. Briefly, Cells were cultured by complete medium containing compounds for 48 h. Then, intracellular melanin was extracted by 1 M NaOH containing 10% DMSO. The absorbance of the dissolved melanin was obtained using a microplate reader at 405 nm. The melanin content of each treatment group was calculated with the negative control group as reference.

#### 4.3.4. Measurement of BTYR Indirectly Inhibitory Activity in Cells Cultured by Compounds

The BTYR indirectly inhibitory activity was assayed according to our previous procedure [19] with slight modification. Cells were cultured by complete medium containing compounds for 48 h and lysed by 120 μL of lysis buffer to obtain crude BTYR extract. The molarities of kojic acid and β-arbutin were 25, 50, 100, 125, 150, 175, 200, 400, 600, 800, 1000 and 1200 μM. The molarities of EGCG were 25, 50, 100, 125, 150, 175 and 200 μM. The treatment group without samples was negative control.

In a reaction system of 100 μL, 20 μL of BTYR was mixed with 60 μL of 8 mM L-DOPA (the remaining volume was supplemented with 20 mM pH 6.8 PBS), and immediately placed at 475 nm for dynamic detection for 30 min. The absorbance was measured once per minute. The control group containing only BTYR (blank control and sample control) was set for synchronous detection to exclude background interference.

With negative control as the reference, the indirectly inhibitory activity of BTYR was calculated in the same way as in Section 4.2.

### 4.4. Chromatic Aberration of Fresh-Cut Apple Slices Soaked by Samples

Colorimetric measurements were carried out using Shanxi Fuji apples, following the procedure of [8] with slight modification. Apples were purchased from the local market with similar size and without injuries, diseases, or pests. The selected apples were washed with distilled water, peeled and cut into 3.5 mm slices using a sharp knife. The starting state of color was fresh-cut apple slice and recorded as 0 h. Then, fresh-cut apple slices were immersed in various test solutions for 0.17 h (10 min), followed by drained and stored at room temperature on a double layer nylon strainer in fume hood without blowing for another 0, 4, 8, and 24 h, recorded as 0.17, 4.17, 8.17 and 24.17 h, respectively. The test solutions were water (negative control groups), kojic acid (1 mM), β-arbutin (1 mM), and EGCG (1 mM). Apple slices without soaking were used as blank control. The processed apple slices were photographed at the experimental time point of each setting. L* (lightness), a* (red-green), and b* (yellow-blue) were measured by a colorimetric spectrophotometer (CM-36 dG spectrophotometer) and were carried out on three replicate slices, in which each slice was recorded four times in distinct parts. This experiment was repeated three times independently. Total color difference (ΔE) was calculated as follows:

$$\Delta E={\left[{\left(L_{t}^{*}-L_{initial}^{*}\right)}^{2}+{\left(a_{t}^{*}-a_{initial}\right)}^{2}+{\left(b_{t}^{*}-b_{initial}\right)}^{2}\right]}^{0.5}\;$$

### 4.5. ATYR Extract Preparation

The preparation of fresh-cut apple slices was the same as in Section 4.4. The preparation of crude ATYR was carried out by reference [41] and slightly adjusted. (1) Fresh-cut apple slice was used to extract enzyme and studied ATYR directly inhibitory activity of compounds. (2) As for indirectly inhibitory activity of compounds, fresh-cut apple slices were immersed in various test solutions for 10 min, followed by rinsed twice with pure water to remove the sticking compounds and then dried with a filter paper. Finally, these apple slices were used to extract ATYR.

20 g of pitted apple slices (about two apple slices) were weighed into a juicer (Bear Electric Appliance Co., Ltd., Foshan, China) containing insoluble PVPP (2 g) and pre-cooling phosphate buffer (PBS, 30 mL, 20 mM, pH 6.8), followed by homogenized for 1 min and centrifuged at 12,000 rpm for 10 min at 4 °C. After the supernatant was divided, it was quick-frozen with liquid nitrogen, and then stored in the refrigerator at −80 °C. The supernatant was used for ATYR activity analysis.

### 4.6. ATYR Directly Inhibitory Activity of Compound

In a reaction system of 200 μL, 40 µL ATYR was mixed with 40 µL corresponding compound in 96-well plates. After incubated at 25 °C for 10 min, L-DOPA (120 µL, 8 mM) was added to each well as a substrate. Once the substrate was added in reaction system, the formation of dopachrome was immediately measured at 475 nm for 30 min at 1 min intervals. The molarities of kojic acid were 0.0125, 0.025, 0.05, 0.1, 0.2, 0.4, 0.8, 1.6, 2.4, 3.2 and 4 mM. The molarities of β-arbutin were 0.025, 0.05, 0.1, 0.2, 0.4, 0.8, 1.6, 3.2, 4, 5, 10, 20, 40, 80, 160 and 320 mM. The molarities of EGCG were 0.00004, 0.0002, 0.001, 0.005, 0.0125, 0.025, 0.05, 0.1, 0.2, 0.4, 0.8, 1.6, 2.4, 3.2 and 4 mM. The group setting and the calculation of ATYR directly inhibitory activity were the same as in Section 4.2.

### 4.7. ATYR Indirectly Inhibitory Activity of Compound

Apple slices, immersing in various test solutions for 10 min, were used to extract crude ATYR solution. The test solutions were water (negative control groups), kojic acid (1 mM), β-arbutin (1 mM), and EGCG (1 mM).

In a reaction system of 200 µL, 40 µL of ATYR was mixed with 120 µL of 8 mM L-DOPA (the deficient part of the system was supplemented with 20 mM pH 6.8 PBS), and the absorbance was measured once per minute at 475 nm for 30 min. The control group containing only ATYR (blank control and sample control) was set for synchronous detection to exclude background interference. With negative control as the reference, the indirectly inhibitory activity of ATYR was calculated in the same way as in Section 4.2.

### 4.8. MTYR Directly Inhibitory Activity of Compound

The MTYR directly inhibitory activity was assayed according to our previous procedure [16]. Briefly, the reaction media was 200 µL in 96-well plates. MTYR (40 µL, 30 U/mL) in PBS (20 mM, pH 6.8) was incubated with the corresponding compound (40 µL) at 25 °C for 10 min. Then, L-DOPA (40 µL, 8 mM) was added to each well as a substrate. The other steps and the calculation of MTYR directly inhibitory activity were the same as above method in Section 4.2.

The molarities of kojic acid were 0.00004, 0.0002, 0.001, 0.005, 0.025, 0.05, 0.1, 0.2 and 0.4 mM. The molarities of β-arbutin were 0.03, 0.06, 0.12, 0.24, 0.48, 0.96, 2, 4, 5, 8, 10, 20, 40, 80, 160 and 320 mM. The molarities of EGCG were 0.00004, 0.0002, 0.001, 0.005, 0.025, 0.1, 0.2, 0.4, 0.8 and 1.6 mM.

### 4.9. qPCR Analysis of TYR Gene

Measurement of mRNA expression of TYR was determined according to our previous procedure [19]. Briefly, B16F10 cells were pretreated with EGCG and kojic acid for 2 h and stimulated with 100 nM α-MSH for 24 h in the presence of EGCG and kojic acid in 6-well cell culture plates (2 mL, 1 × 10^5^ cells/mL). Total RNAs prepared were followed and qPCR analysis of TYR were carried on with β-actin as an internal control.

### 4.10. Kinetic Analysis of MTYR

The experimental method was the same as Section 4.8, except for the substrate concentration and compound concentration. A series of experiments were performed to determine the type of inhibition.

The molarities of L-DOPA were 0.0625, 0.25, 1, 4 and 8 mM. The molarities of kojic acid were 25, 50 and 100 µM, while that of EGCG were 200, 400 and 800 µM. The *K_m_* and *V_max_* of MTYR were determined by the Michaelis–Menten equation. The Lineweaver–Burk plot was used to display the data. Secondary replot of Lineweaver–Burk plot or intercept was used to display inhibition constant (Ki) and α.

### 4.11. Statistical Analysis

All experiments were carried out at least in three independent trials. The experimental data were expressed as the mean ± standard deviation. One-way analysis of variance was carried out by SPSS 21.0 (SPSS Inc., Chicago, IL, USA) and was used to compare the value differences (*p* < 0.05). Michaelis–Menten equation and Lineweaver–Burk plot were analyzed and drawn using GraphPad Prism (Version 9.00, GraphPad Software Inc., San Diego, CA, USA).

## 5. Conclusions

In summary, this study found that the TYR inhibitory activity of samples was affected by the species of enzyme and the sample addition methods. In view of the important effect of enzyme species on the TYR inhibitory activity of samples, it is necessary to select the appropriate enzyme according to the problems to be explained. In addition, the results of indirectly inhibitory activity reflect the comprehensive effects of samples on TYR catalytic activity and intracellular TYR synthesis pathway, while the results of directly inhibitory activity mainly reflect the effects of samples on TYR catalytic activity. Kojic acid directly inhibited the activity of BTYR and MTYR in a dose-dependent manner, and kojic acid also indirectly inhibited the activity of BTYR in a dose-dependent manner. Therefore, kojic acid could be used as a positive control for both B16F10 cells and MTYR models. In terms of directly inhibitory effect, β-arbutin could inhibit or promote TYR activity according to different species of TYR, or even promote TYR activity first and then inhibit TYR activity in a dose-dependent manner. Therefore, the directly inhibitory activity of β-arbutin on TYR was unstable. β-arbutin could indirectly inhibit the activities of BTYR and ATYR. In directly inhibitory activity, EGCG maintained a stable inhibitory activity on TYR of different species. EGCG also indirectly inhibited BTYR activity in a dose-dependent manner. It could be speculated that the TYR inhibitory activity of EGCG is universal. Compared with the unstable β-arbutin, EGCG might be more valuable for clinical research.

## Figures and Tables

**Table 1:** Cell viability under each compound treatment.

Molarity (µM)	Cell Viability (%)		
	Kojic Acid	β-Arbutin	EGCG
1200	98 ± 2	222 ± 2	−1 ± 0
1000	98 ± 4	211 ± 1	1 ± 1
800	99 ± 4	206 ± 0	2 ± 1
600	96 ± 5	195 ± 3	4 ± 0
400	90 ± 3	171 ± 1	66 ± 4
200	90 ± 3	138 ± 3	106 ± 1
100	95 ± 1	125 ± 2	95 ± 6
50	101 ± 2	115 ± 4	98 ± 2
25	100 ± 4	108 ± 2	100 ± 2

## Data Availability

The data are contained within the article and supplementary material.

## Supplementary Materials

The following supporting information can be downloaded at: https://www.mdpi.com/article/10.3390/ijms24076013/s1.

## References

[1] Mayr F., Sturm S., Ganzera M., Waltenberger B., Martens S., Schwaiger S., Schuster D., Stuppner H. (2019). Mushroom tyrosinase-based enzyme inhibition assays are not suitable for bioactivity-guided fractionation of extracts. J. Nat. Prod..

[2] Kupper U., Niedermann D.M., Travaglini G., Lerch K. (1989). Isolation and characterization of the tyrosinase gene from Neurospora crassa. J. Biol. Chem..

[3] van Gastel M., Bubacco L., Groenen E.J., Vijgenboom E., Canters G.W. (2000). EPR study of the dinuclear active copper site of tyrosinase from Streptomyces antibioticus. FEBS Lett..

[4] Zhao M.J., Hu J.J., Ni H., Jiang Z.D., Wang L. (2019). Research progress in melanogenesis signaling pathway. Chin. J. Biotechnol..

[5] Seo S., Sharma V.K., Sharma N. (2003). Mushroom tyrosinase: Recent prospects. J. Agric. Food Chem..

[6] Martinez M.V., Whitaker J.R. (1995). The biochemistry and control of enzymatic browning. Trends Food Sci. Technol..

[7] Zolghadri S., Bahrami A., Hassan Khan M.T., Munoz-Munoz J., Garcia-Molina F., Garcia-Canovas F., Saboury A.A. (2019). A comprehensive review on tyrosinase inhibitors. J. Enzyme Inhib. Med. Chem..

[8] Carcelli M., Rogolino D., Bartoli J., Pala N., Compari C., Ronda N., Bacciottini F., Incerti M., Fisicaro E. (2020). Hydroxyphenyl thiosemicarbazones as inhibitors of mushroom tyrosinase and antibrowning agents. Food Chem..

[9] Pei C.J. (2013). Inhibitory Effects of Gartrodin on Tyrosinase and Melanogenesis. Master’s Thesis.

[10] Sun X., Qiao Q.A., Liu C.C., Jiang H.H. (2016). Research progress on the mechanisms of tyrosinase catalyzed reactions. J. Ludong Univ. (Nat. Sci. Ed.).

[11] Strzępek-Gomółka M., Gaweł-Bęben K., Angelis A., Antosiewicz B., Sakipova Z., Kozhanova K., Głowniak K., Kukula-Koch W. (2021). Identification of mushroom and murine tyrosinase inhibitors from achillea biebersteinii afan. Extract. Molecules.

[12] Bouhoute M., Amen Y., Bejaoui M., Mizushima A.K.O., Shimizu K., Isoda H. (2022). New butyroside D from Argan press cake possess anti-melanogenesis effect via MITF downregulation in B16F10 and HEM cells. Int. J. Mol. Sci..

[13] Lim S.J., Jung D.W., Hillman P.F., Nam S.J., Lee C.S. (2022). SNA077, an extract of marine *Streptomyces* sp., inhibits melanogenesis by downregulating melanogenic proteins via inactivation of cAMP/PKA/CREB signaling. Int. J. Mol. Sci..

[14] Funayama M., Arakawa H., Yamamoto R., Nishino T., Shin T., Murao S. (1995). Effects of alpha- and beta-arbutin on activity of tyrosinases from mushroom and mouse melanoma. Biosci. Biotechnol. Biochem..

[15] Sugimoto K., Nishimura T., Nomura K., Sugimoto K., Kuriki T. (2003). Syntheses of arbutin-alpha-glycosides and a comparison of their inhibitory effects with those of alpha-arbutin and arbutin on human tyrosinase. Chem. Pharm. Bull..

[16] Wang W., Gao Y., Wang W., Zhang J., Yin J., Le T., Xue J., Engelhardt U.H., Jiang H. (2022). Kojic acid showed consistent inhibitory activity on tyrosinase from mushroom and in cultured B16F10 cells compared with arbutins. Antioxidants.

[17] Pillaiyar T., Manickam M., Namasivayam V. (2017). Skin whitening agents: Medicinal chemistry perspective of tyrosinase inhibitors. J. Enzyme Inhib. Med. Chem..

[18] Fu D., Yuan Y., Qin F., Xu Y., Cui X., Li G., Yao S., Deng Y., Tang Z. (2021). Design, synthesis and biological evaluation of tyrosinase-targeting PROTACs. Eur. J. Med. Chem..

[19] Wang W., Chen L., Wang W., Zhang J., Engelhardt U.H., Jiang H. (2022). Effect of active groups and oxidative dimerization on the antimelanogenic activity of catechins and their dimeric oxidation products. J. Agric. Food Chem..

[20] Chen J., Ran M., Wang M., Liu X., Liu S., Ruan Z., Jin N. (2022). Evaluation of antityrosinase activity and mechanism, antioxidation, and UV filter properties of theaflavin. Biotechnol. Appl. Biochem..

[21] He M., Fan M., Liu W., Li Y., Wang G. (2021). Design, synthesis, molecular modeling, and biological evaluation of novel kojic acid derivatives containing bioactive heterocycle moiety as inhibitors of tyrosinase and antibrowning agents. Food Chem..

[22] Mann T., Gerwat W., Batzer J., Eggers K., Scherner C., Wenck H., Stab F., Hearing V.J., Rohm K.H., Kolbe L. (2018). Inhibition of human tyrosinase requires molecular motifs distinctively different from mushroom tyrosinase. J. Investig. Dermatol..

[23] Hearing V.J., Ekel T.M., Montague P.M., Nicholson J.M. (1980). Mammalin tyrosinase. Stoichiometry and measurement of reaction products. Biochim. Biophys. Acta..

[24] Roulier B., Pérès B., Haudecoeur R. (2020). Advances in the design of genuine human tyrosinase inhibitors for targeting melanogenesis and related pigmentations. J. Med. Chem..

[25] Gregus Z., Madhu C., Klaassen C.D. (1988). Species variation in toxication and detoxication of acetaminophen in vivo: A comparative study of biliary and urinary excretion of acetaminophen metabolites. J. Pharmacol. Exp. Ther..

[26] Shen Y., Wang L.N., Yin Z.F. (2020). Effect of species difference of experimental animals on traditional chinese medicine research. Chin. Med. Mod. Distance Educ. China.

[27] Liu S.T., Li J.M., Si D.Y., Li X.Y. (1995). An in vitro study of cortex sophorae extract on intestine and uterus muscles of animals. J. Jining Med. Coll..

[28] Sugimoto K., Nishimura T., Nomura K., Sugimoto K., Kuriki T. (2004). Inhibitory effects of alpha-arbutin on melanin synthesis in cultured human melanoma cells and a three-dimensional human skin model. Biol. Pharm. Bull..

[29] Chakraborty A.K., Funasaka Y., Komoto M., Ichihashi M. (1998). Effect of arbutin on melanogenic proteins in human melanocytes. Pigm. Cell Melanoma Res..

[30] Nakajima M., Shinoda I., Fukuwatari Y., Hayasawa H. (1998). Arbutin increases the pigmentation of cultured human melanocytes through mechanisms other than the induction of tyrosinase activity. Pigm. Cell Melanoma Res..

[31] Cell Counting Kit-8. https://www.beyotime.com/product/C0039.htm.

[32] Xu H., Li X., Xin X., Mo L., Zou Y., Zhao G., Yu Y., Chen K. (2021). Antityrosinase mechanism and antimelanogenic effect of arbutin esters synthesis catalyzed by whole-cell biocatalyst. J. Agric. Food Chem..

[33] Xiong S.L., Lim G.T., Yin S.J., Lee J., Si Y.X., Yang J.M., Park Y.D., Qian G.Y. (2019). The inhibitory effect of pyrogallol on tyrosinase activity and structure: Integration study of inhibition kinetics with molecular dynamics simulation. Int. J. Biol. Macromol..

[34] Muñoz J.L., García-Molina F., Varón R., Rodriguez-Lopez J.N., García-Cánovas F., Tudela J. (2006). Calculating molar absorptivities for quinones: Application to the measurement of tyrosinase activity. Anal. Biochem..

[35] Muñoz-Muñoz J.L., García-Molina F., García-Ruiz P.A., Molina-Alarcón M., Tudela J., García-Cánovas F., Rodríguez-López J.N. (2008). Phenolic substrates and suicide inactivation of tyrosinase: Kinetics and mechanism. Biochem. J..

[36] Chen Q.X., Kubo I. (2002). Kinetics of mushroom tyrosinase inhibition by quercetin. J. Agric. Food Chem..

[37] Kubo I., Nihei K., Shimizu K. (2004). Oxidation products of quercetin catalyzed by mushroom tyrosinase. Bioorg. Med. Chem..

[38] Lü Z., Shi L., Wang J., Park D., Bhak J., Yang J.-M., Park Y., Zhou H., Zou F. (2010). The effect of trifluoroethanol on tyrosinase activity and conformation: Inhibition kinetics and computational simulations. Appl. Biochem. Biotechnol..

[39] Wang Z.J., Si Y.X., Oh S., Yang J.M., Yin S.J., Park Y.D., Lee J., Qian G.Y. (2012). The effect of fucoidan on tyrosinase: Computational molecular dynamics integrating inhibition kinetics. J. Biomol. Struct. Dyn..

[40] Qin L., Wu Y., Liu Y., Chen Y., Zhang P. (2014). Dual effects of alpha-arbutin on monophenolase and diphenolase activities of mushroom tyrosinase. PLoS ONE.

[41] Hou Z., Feng Y., Wei S., Wang Q. (2014). Effects of curing treatment on the browning of fresh-cut potatoes. Am. J. Potato Res..

