# Supplementary material for: Inhibitory Activities of Samples on Tyrosinases Were Affected by Enzyme Species and Sample Addition Methods
Source: Int J Mol Sci. 2023 Mar 23;24(7):6013. doi: 10.3390/ijms24076013 (PMC10093845; doi:10.3390/ijms24076013)
Supplement: Supplementary file 1 [file ijms-24-06013-s001.zip › ijms-2222256-supplementary.pdf]

Supplementary Material

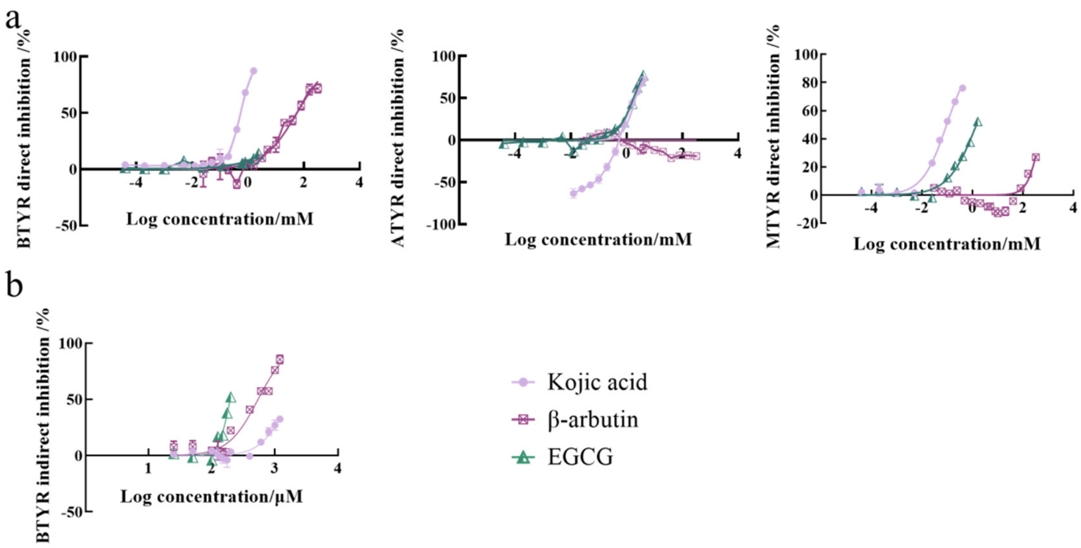

Figure S1. EC_{50} curves of TYR inhibition activity of samples

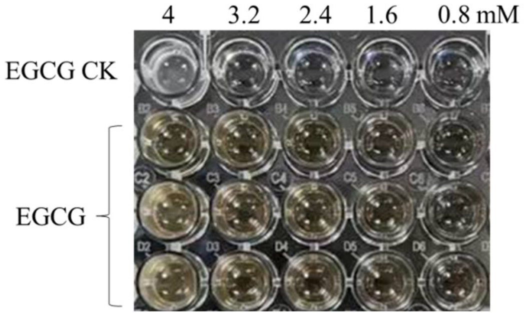

Figure S2. High concentration of epigallocatechin gallate (EGCG) complexed with BTYR.

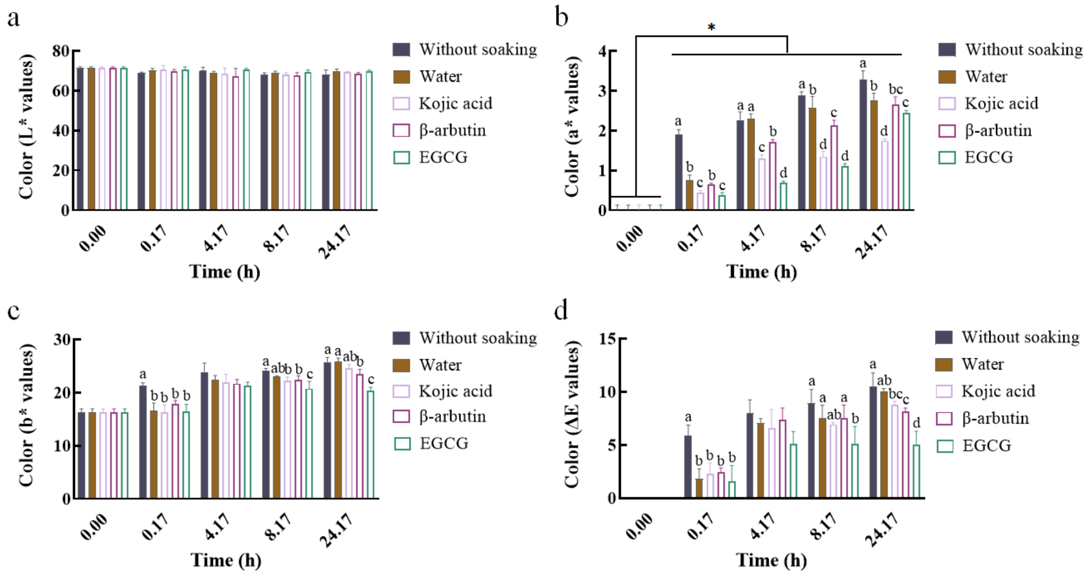

Figure S3. Comparison of chromatic value of each treatment at the same time point. (a) L* value difference; (b) a* value difference; (c) b* value difference; (d) ΔE value difference. [*] There being significant difference compared with fresh-cut apples (0 h, $p < 0.05$). [a,b,c,d] Different letters above the plots indicate significant differences among different treatments at the same time ($p < 0.05$).
